# Monitoring health and wellbeing in adolescent track and field (athletics) athletes: A co-creation study

**DOI:** 10.1371/journal.pone.0341972

**Published:** 2026-02-27

**Authors:** Natalie A. Bunce, Jo Day, Robert H. Mann, Anna Jennings, Alan R. Barker

**Affiliations:** 1 Children’s Health and Exercise Research Centre, Department of Public Health and Sport Sciences, Faculty of Health and Life Sciences, University of Exeter, Exeter, United Kingdom; 2 NIHR Applied Research Collaboration South West Peninsula, Department of Health and Community Sciences, Faculty of Health and Life Sciences, University of Exeter, Exeter, United Kingdom; 3 Podium Analytics, London, United Kingdom; Portugal Football School, Portuguese Football Federation, PORTUGAL

## Abstract

Talent Development Environments, such as England Athletics’ Youth Talent Programme (YTP), aim to provide holistic support that promotes long-term athletic and personal development. There is a need for National Governing Bodies to understand athlete health and wellbeing (HWB) to increase athlete availability, wellbeing, and performance. Regarding the YTP, this study aimed to: (1) understand HWB concepts and current monitoring practices; (2) explore design preferences for athlete HWB monitoring; and (3) identify factors that influence the use and implementation of a HWB monitoring system. An online survey was completed by 53 YTP athletes (31 female) capturing demographics, HWB understanding, current monitoring behaviours, and design preferences. Six focus groups and one interview were subsequently conducted with YTP staff (n = 11) and athletes (n = 8). Staff discussions explored current data collection methods, factors that influence system adoption, and integration possibilities. Athlete discussions explored HWB attitudes, factors that influence system adoption, and design preferences. Survey findings indicated that athletes regard monitoring HWB as ‘important’ (36%) or ‘very important’ (26%). However, 62% of athletes do not keep a training diary and 45% do not monitor their HWB, confirming they lack tools and/or knowledge to monitor effectively. Sixty-six percent of athletes who do not monitor would like to improve HWB knowledge. Among those who monitor (55%), the most frequently recorded were training (n = 19) and sleep (n = 14) data. Reflexive thematic analysis generated four interrelated themes: (1) *education as a pathway to athlete autonomy and wellbeing*; (2) *holistic HWB perspectives*; (3) *monitoring practices*; and (4) *factors that influence adoption.* Findings highlight the importance of HWB education and whole person development. Despite limited formal monitoring knowledge, athletes engage in self-monitoring and are willing to improve HWB knowledge. Preference for mobile accessibility, adaptability, and reminders, together with socio-environmental factors, will inform the design of an athlete HWB monitoring system for the YTP.

## Introduction

The Youth Talent Programme (YTP) is the first step on England Athletics’ talent pathway – designed to support identified talented athletics (track and field) athletes aged 16–18 years. As a Talent Development Environment [1], the YTP provides a holistic programme that educates athletes on psychological, environmental, and lifestyle behaviours, in addition to physical preparation. The YTP helps athletes develop the skills and knowledge in a performance environment required to pursue a dual career. This prepares athletes for transition along the talent pathway or employment within the wider high performance sector [2]. This approach aims to support athletes in attaining successful, sustainable dual careers [3–5], develop a self-determined involvement in sport on graduation from the talent pathway [6], and maintain a healthy lifestyle outside of sport [4,7].

Adolescent athletes are at risk of increased health problems (i.e., injury and illness) and negative wellbeing [8–12]. Both as an athlete and young person, they are navigating increasingly structured training practice [13] and psychosocial stressors, including academic [8], peer, and/or family pressures [14] which can fluctuate throughout a calendar year [15]. How these stressors are managed in conjunction with athletics training play a vital role in the athletes overall health and wellbeing (HWB), their continuation along the talent pathway, and/or sustained participation in sport as an adult. Consequently, Talent Development Environments should aim to create conditions that promote an athlete’s long-term personal development, wellbeing, and athletic development. These conditions concern the physical environment (e.g., system-based support and skilled/capable staff), a culture of athlete empowerment, psychological safety, and collaboration with stakeholders outside of the sport [6]. This emphasises that Talent Development Environments have a responsibility beyond elite level performance to support continued involvement in sport for an active lifestyle [6]. Currently, the YTP does not have a complete picture of the HWB of enrolled athletes, which could be used to further support personal development and wellbeing. One way in which the YTP can start to understand athlete HWB is by implementing appropriate monitoring systems.

Athlete monitoring systems typically comprise of objective (e.g., performance, physiological, biochemical) [16] and subjective (including perceptions of wellbeing and psychological variables) measures [17]. Subjective measures, usually captured via self-report surveys [18–21], can identify subtle changes in athlete HWB in comparison to objective measures [16]. Brief, daily or weekly self-report measures, supplemented with a more in-depth measure periodically may be sufficient to assist in the athlete become more self-aware in their rest and recovery needs [22]. Monitoring systems have been successful in team sports, such as football and rugby union, where the team environment aids data capture as athletes are situated in one place and train together, making it logistically easier to ensure compliance and consistency of data collection with coaches being able to facilitate this [23,24]. However, in an athletics development environment, such as the YTP, the individualisation of disciplines (i.e., endurance, jumps, throws, sprints, combined events) may require different measures of training load. Furthermore, athletes are distributed throughout the country, training in separate athletic clubs and are coached individually by different coaches resulting in differing training practices. The athletes only meet occasionally as a whole group for training camps; this makes the collection of HWB data more complex within the YTP compared to team sports.

The way monitoring systems employing self-report measures are used and implemented can influence the effectiveness of the data collected [25]. Some measures require daily reporting for acute changes, or weekly/monthly reporting [25–27] for longer term outcomes [25]. Sport organisations have invariably adopted single-item scales [28] or have customised surveys, made up of multiple single item questions [29]. Other challenges with self-report measures include miscomprehension and recall error [30], in addition to requiring multi-level implementation strategies [31]. This highlights the importance of both system design and consideration of social-ecological factors [28].

To increase the chance of monitoring system adoption, multi-level buy-in [28] is required. A co-creation approach, enabling academics to work alongside project partners to develop the system [32], is a way of overcoming the knowledge-to-practice gap. This assists in creating more relevant, acceptable, and usable interventions in the setting for which it is intended [33].

**Fig 1:**
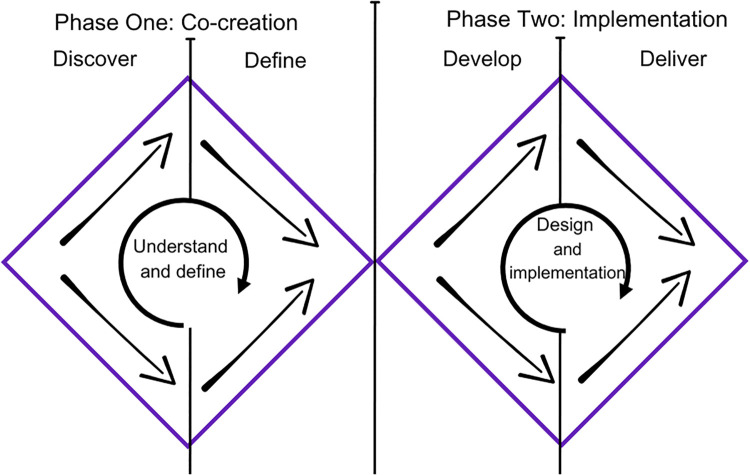
The Double Diamond Framework. The Double Diamond Framework demonstrating the monitoring system design process, adapted from The Design Council [34].

This study represents phase one of a larger research project aimed at co-creating (phase one) and implementing (phase two) a monitoring system to understand HWB prevalence of adolescent athletes. Both phases are guided by Double Diamond Framework [34] which incorporates divergent (creative) and convergent (logic) thinking to support collaborative exploration when designing an innovation. The framework has been successfully used to co-develop a whole school physical activity framework [35] and to co-design service improvements in health and social care settings [36]. In phase one, the Discovery and Define stages (Fig 1) are used to explore athlete and staff views on HWB concepts and monitoring. Insights from phase one will inform the design of the system for phase two, where the Develop and Deliver stages will be utilised. This represents a novel approach to creating and implementing a HWB monitoring system for adolescent athletes in a Talent Development Environment.

The broader project aims to: (1) produce a dataset to identify the HWB prevalence of athletes on the YTP; (2) increase athlete self-awareness and autonomy about their own HWB; (3) understand injury and illness across athletics event groups to inform future interventions; and (4) understand athlete wellbeing to inform future intervention development and support junior-to-senior pathway transitions.

The purpose of phase one of this project was to undertake initial exploratory work to identify considerations and potential challenges surrounding the creation of an athlete monitoring system within the YTP context. To define the design and implementation strategy for the proposed monitoring system, this study aimed to explore YTP staff and athlete:

(1) understanding of HWB concepts and current monitoring practices;
(2) system design preferences, exploring the content, form, and delivery method; and
(3) perceived factors that influence the use and implementation of a monitoring system within the YTP.

## Methodology

### Study design

The researchers adopted a pragmatic approach, incorporating both quantitative and qualitative methods. This flexible approach enabled collaboration between researchers, stakeholders and end users in exploring HWB concepts, organisational integration of the monitoring system and system design preferences during the creation of the monitoring system. Phase one employed a cross-sectional design and was split into two successive parts. Part one involved completion of an online survey (athletes) and part two consisted of focus groups (athletes and staff) and a supplementary interview (staff). Using a mixed methods approach allowed the researchers to gather standardised numerical data and brief qualitative insights through survey responses from a larger number of athletes which was complemented by rich, contextualised insights from the focus groups to improve understanding to the survey findings [37]. Ethics approval was granted by the University of Exeter Public Health and Sport Sciences Ethics Committee (Approval Reference 6284286).

### Part one: online survey

#### Participant characteristics.

A purposive sampling technique was used to invite 16–18-year-old athletes who, at the time of data collection were enrolled on the YTP. Recruitment for the online survey was initiated by relevant YTP administrative staff, as gatekeepers, who emailed all YTP athletes. The recruitment email contained a participant information sheet which set out the study aims, methods, benefits, and risks associated with taking part in the study. Due the age of the participants (16–18 year old), no parental consent was required [38]. Athletes were advised to discuss their taking part in the study with parents/carers before starting the survey. The participants were also encouraged to contact the research team with any questions they may have. The recruitment email contained a link to the survey. Before starting the survey, athletes provided electronic written consent.

Overall, 53 YTP athletes enrolled on the YTP (at the time of data collection) completed the survey. Thirty-one (58%) participants were female and 22 (42%) males. 55% (n = 29) of athletes selected “endurance” as their main event group and 81% (n = 43) of athletes competed at National Level which was defined as “track and field championships, cross country championships, road championships, and UK School Games provided by England Athletics”. Survey participant characteristics are shown in Table 1.

**Table 1:** Survey Participants Characteristics.

Characteristics	Athletes (n = 53)	%
**Event Group**		
Endurance	29	55
Throws	9	17
Sprints	7	13
Combined Events	5	9
Jumps	3	6
**Competition Level**		
International Level	9	17
National Level	43	81
Regional Level	1	2
**Years competing in athletics**		
1–2 years	8	15
3–4 years	14	26
5–6 years	17	32
7–8 years	13	25
More than 10 years	1	2
**Years taking part in athletics**		
1–2 years	9	17
3–4 years	11	21
5–6 years	15	28
7–8 years	13	25
9–10 years	1	1
More than 10 years	4	8
**Weekly training hours in main event**		
1–2 hours	2	4
3–4 hours	10	19
5–6 hours	14	26
7–8 hours	8	15
9–10 hours	11	21
More than 10 hours per week	8	15

#### Methods.

The survey questions (S1 File) were developed based on literature indicating that system design features such as single item measures, accessibility, reminders and contextual factors including athlete feedback, motivation and time constraints can influence adoption of athlete monitoring systems [25,39,40].The survey was designed using Qualtrics XM (Utah, USA) and contained Likert scale, ranking, and open and closed questions and was split into three parts: (1) background demographics and training history; (2) understanding of HWB and current monitoring behaviours; and (3) user preferences in relation to design and delivery of a monitoring system. The length of the survey was designed to take between 20 and 30 minutes to complete.

Prior to distribution to participants, the survey was reviewed for face validity by five reviewers (four academics in sport and health sciences and one reviewer in research and innovation specialising in youth sport environments) and four adolescents, two of which participate in athletics. Each reviewer completed a face validity review asking questions relating to clarity, ambiguity, reasonability, and relevancy of the survey [41]. Following the review, the survey was modified to include definitions to add clarity and aid question understanding for the age of the participants. The survey was the checked for readability producing a Flesch reading ease score of 69.4, confirming the readability of the survey was appropriate for individuals 16–18 year olds.

The survey provided athletes with definitions of physical health, mental health, and social wellbeing. Physical health was framed as a participant having no injury/illness. Mental health meant that a participant could cope with the normal stressors of life, whilst social wellbeing was defined as a participant who can maintain and build healthy relationships with family, friends, and colleagues [42]. As part of the survey, athletes were provided with a list of HWB items and asked to rate each item using a 6-point Likert being ‘Very important’, ‘Important’, ‘Moderately Important’, ‘Somewhat Important’, ‘Not Important’ and ‘Don’t know’ (S1 File). For analysis, numerical values were assigned to each response option, with ‘Very important’ scored as 6 and ‘Don’t know’ scored as 1. The scores for each item were then summed.

The survey was first circulated via email by an England Athletics’ administrator, to all first-year athletes (n = 242) enrolled on the YTP, on 29/09/2024. Additional recruitment was conducted across both cohorts (year one and year two) on 12/10/2024 and 26/10/2024 as part of in-person YTP activities (i.e., camp days) where they were provided with a QR code to access the survey. The survey was open for 47 days and closed on 08/11/2024. Participants completed the survey in an average of 22.9 minutes (interquartile range 25% − 75%: 15.9–28.6 minutes).

#### Statistical analysis.

Raw survey data was downloaded into a Microsoft Excel spreadsheet and manually scanned for missing or incorrect data. A total of 90 incomplete responses were found of which 39 participants exited before providing consent and starting the survey, nine participants provided their consent but did not start the survey and 42 participants exited at different points throughout the survey. Incomplete data were deleted from the data set.

Quantitative survey data were analysed using Microsoft Excel and IBM SPSS [29.0.1.0] to show frequencies and percentages and qualitative data were analysed with focus groups data to form one data set using reflexive thematic analysis following the six phases detailed by Braun & Clarke [43]

### Part two: focus groups and interview

#### Participant characteristics.

For the athlete focus group, at the end of the survey, athletes were asked to tick a box if they would like to take part in a focus group relating to the topics contained in the survey. Athletes who confirmed they would like to participate in the focus group also provided their name and email address for the research team to contact them direct. Following completion of the survey, all athletes who provided their name and email address (n = 19) were contacted by the research team and invited by email to take part in the focus groups. After being contacted, 10 athletes failed to respond, and one athlete consented to take part but failed to attend the focus groups.

For the staff focus groups, all YTP coaches, event group leaders and management staff were initially emailed by relevant England Athletics’ administrative staff involved in the YTP, the email contained details of the study and asked for consent for the research team to contact willing participants for recruitment. For potential participants (n = 20) who provided their consent, the research team contacted them by email to recruit for relevant focus groups. Where it is was not possible for a member of YTP staff to attend a focus group, an individual interview was offered to ensure that all relevant views are captured. After being contacted, eight staff members failed to respond and one staff member consented to take part but could not attend any focus groups.

Recruitment emails to all focus groups contained a participant information sheet and a link to an online consent form which was completed before the start of the focus group. At the start of each focus group/interview, verbal consent was further confirmed by each participant to take part understanding that their participation is voluntarily and reminded about their right to withdraw at any time.

Eight athletes (5 female) representing jumps (n = 1), throws (n = 1), combined events (n = 2), and endurance (n = 4) and ten staff members attended the focus groups (of which YTP coaches and event group leads represented – throws, jumps, and endurance event groups) and one staff member attended an interview.

#### Method.

A semi-structured approach was used adopting topics guides to encourage interaction to illicit natural responses and capture participants unique perspectives and expertise [44]. Results of the survey informed the design of the topic guides and inform discussion of the focus groups/interview. The interview questions followed the focus group topic guides with minor adaptation. The topic guides were designed with the study aims in mind and were divided into three parts: part one discussed HWB concepts; part two asked questions relating to participant’s experience of using monitoring systems; and part three related to system design and preferences, for example platforms, frequency, and duration of data input (S2 File).

Following the closure of the survey, recruitment for the focus groups/interviews started on 08/11/2024. The focus groups (n = 6) and interview (n = 1) took place remotely via Microsoft Teams throughout November and December 2024, and were audio and video recorded. Each focus group lasted between 60–90 minutes and the interview lasted 60 minutes. Digital recordings of focus groups/interviews were transcribed verbatim in Microsoft Word and pseudonymised. After which digital recordings were deleted.

The objective of the focus groups and interview involving staff was to understand current data collection methods, factors that may influence adoption, and how to integrate the proposed system within the YTP to effectively reach its athletes. The objective of focus groups involving athletes was to build on the athlete survey responses to understand HWB attitudes, identify end-user factors that may influence adoption, and design preferences. To help encourage conversation, the first topic for discussion in each part involved a Microsoft PowerPoint slide, where participants were shown a visual of a finding from the athlete survey; for example, a chart illustrating a list of HWB items ranked in order of importance by athletes. The finding was then discussed between each participant to ascertain their views.

#### Data analysis.

Data were analysed inductively using reflexive thematic analysis following the six phases detailed by Braun and Clarke [43] to gain more in-depth understanding of staff and athlete views. Initially, the seven transcripts were separated to form two groups: (1) staff, and (2) athletes and upon familiarisation of the data, summaries relating to each group were produced. This helped gain a broader understanding of insights relating to each group. However, as the focus groups and interview were largely informed by topic guides (based on study aims) each transcript provided data relating to HWB insights, monitoring practices, factors that influence system adoption, and system design preferences and therefore analysis of common themes and patterns were sought across the whole data set and then comparisons were made across the staff and athlete groups to provide an in depth understanding of their experiences.

**Fig 2:**
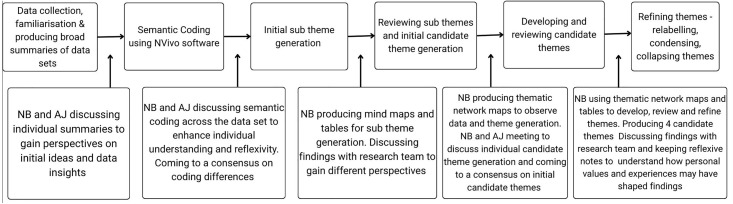
Qualitative Data Analysis Process Chart. Qualitative data analysis process chart informed by the six steps of reflexive thematic analysis, Braun & Clarke [43].

Following familiarisation, transcripts were coded by NB (lead researcher) using NVivo Qualitative Software package [14.23.1(38)]. An experiential qualitative framework was adopted to explore participants meanings, views, perspectives, and experiences. Data were also co-coded by AJ using NVivo and NB and AJ met to discuss their findings relating to semantic codes and to agree on any differences before proceeding with theme generation. As patterns developed across all data sets, NB and AJ individually generated sub themes using mind maps to illustrate findings, these sub-themes then formed potential candidate themes which consisted of descriptive and interpretive themes. Again, following sub-theme generation and subsequently candidate theme generation, NB and AJ met at each phase to discuss findings and agree on differences before proceeding to the next phase. NB created thematic network maps [45] and proceeded to revisit candidate themes and their sub themes, relabelling, condensing or collapsing themes until the final candidate themes formed with their associated sub themes. Following theme reviews and final theme formation, NB and AJ agreed that the themes represented the data. See Fig 2 for the data analysis process.

To improve trustworthiness and rigour of data analysis, NB developed an audit trail for transparency and to note NB reflections. After each analysis phase, NB shared with the wider research team the developing themes to gain different perspectives on the data. The wider research team agreed with theme development throughout analysis. During analysis NB adopted multiple techniques from written summaries, tables, mind maps, thematic networks, and illustrations to interpret the data in multiple ways and maintained a reflexive diary throughout data collection and analysis to note subjective interpretations of the data and preconceptions that may influence findings.

## Results

### Part one: online survey

#### Health and wellbeing.

**Fig 3:**
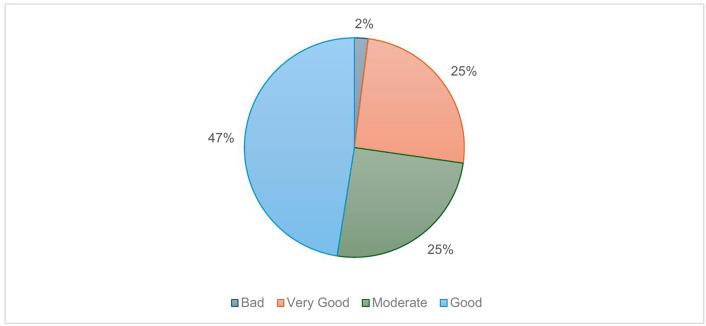
Athlete (n = 53) Health and Wellbeing States. Pie chart showing athlete survey response relating to their current health and wellbeing states (percentages).

**Fig 4:**
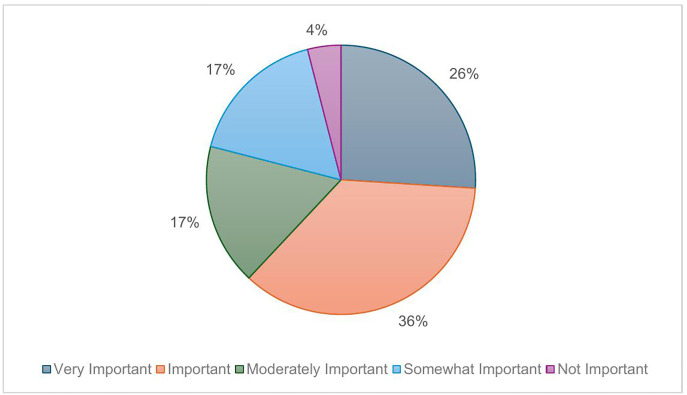
Athlete (n = 53) rating of the importance of monitoring Health and Wellbeing as part of their athletics training. Pie chart showing athlete survey response relating to rating of the importance of monitoring HWB as part of their athletics training.

**Fig 5:**
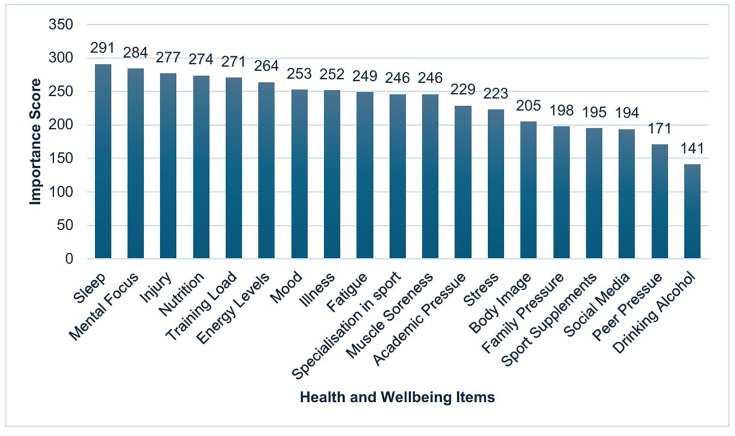
HWB items ranked in order of importance at Youth Talent Programme athlete population level. Bar chart showing survey response (population level) ranking health and wellbeing items in order of importance (summed scores).

47% (n = 25) of athletes self-reported their HWB as “good” with 25% (n = 13) rated as “very good” and 25% (n = 13) as “moderate” (Fig 3). 43% (n = 23) of athletes rated their knowledge of HWB as “good” and 42% (n = 22) as “moderate”. Athletes were asked to rate the importance of monitoring HWB as part of their athletics training and 36% (n = 19) of athletes rated this as “important” and 26% (n = 14) as “very important” (Fig 4). Athletes were also asked to individually score a list of HWB items in terms of their importance. “sleep”, “mental focus”, and “injury” were rated the most important (Fig 5).

#### Current monitoring practices.

**Fig 6:**
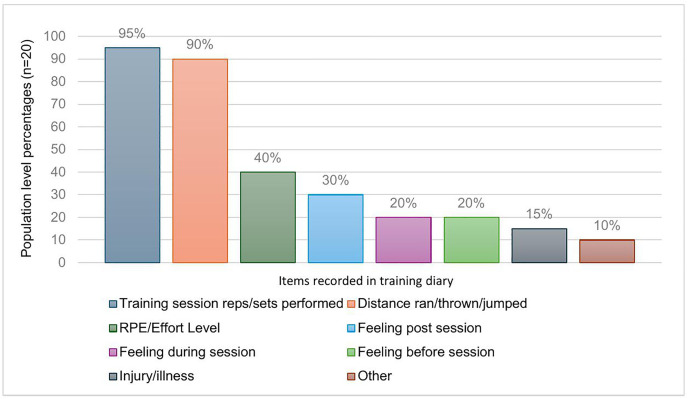
Training information recorded by athletes. Bar chart representing key information recorded by participants who keep a training diary (n = 20) in percentages. Athletes selected multiple options thus each item represents a population percentage.

62% (n = 33) of athletes confirmed that they do not keep a training diary. Athletes who do keep a training diary (n = 20) were asked further questions in relation to their monitoring practices with seven athletes confirming they completed a training diary daily and six athletes completed 1–3 times per week. 50% (n = 10) of athletes spent 0–5 minutes completing their training diary and 55% (n = 11) also used their training diary to monitor their HWB. 60% (n = 12) of athletes completed their training diary after a training session. The most popular items recorded by athletes were how many reps or sets performed during a training session (95%) and distance ran/thrown/jumped (90%), followed by rating of perceived exertion (40%) (Fig 6).

**Fig 7:**
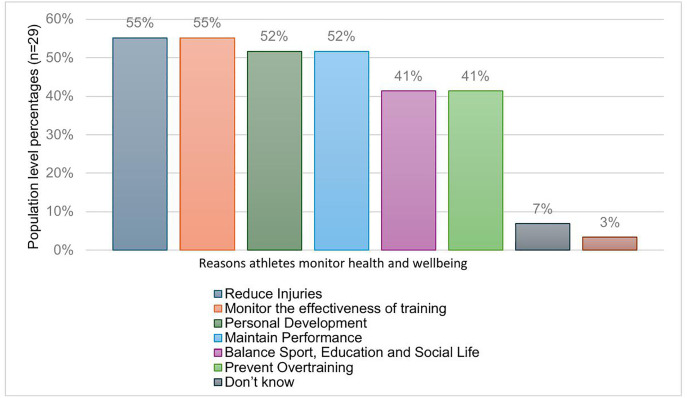
Athlete reasons for monitoring health and wellbeing. Bar chart representing athletes’ reasons for monitoring health and wellbeing (n = 29). Athletes selected multiple option thus each item represents a population percentage.

45% (n = 24) of athletes do not currently monitor their HWB and 42% (n = 22) of athletes sometimes monitor their HWB. For athletes who monitor or sometimes monitor their HWB (n = 29), the most popular recorded items were training (n = 19), sleep (n = 14), and nutrition (n = 7) data. Smart watches and training applications were the most popular method of recording training (n = 11), sleep (n = 10) and nutrition (n = 2) data. Athletes (n = 29) were asked to select their main reasons for monitoring their HWB (multiple choice). The main reasons for monitoring were to reduce injuries, monitor the effectiveness of their training, personal development, and maintain performance (Fig 7). 42% (n = 10) of athletes who do not currently monitor stated that they do not have the tools or know how to monitor. 66% (n = 16) of athletes who confirmed they do not monitor also reported that they would like to improve their knowledge of HWB.

#### User preferences.

28% (n = 15) of athletes were willing to record their HWB 1–3 times per week, 25% (n = 13) daily and 23% (n = 12) once per week. Irrespective of whether athletes were willing to record daily, 1–3 times per week or weekly, it was indicated that athletes were willing to spend 5–10 minutes per day (n = 6)/ week (n = 13) recording. 58% (n = 31) athletes indicated that they would like to receive recording reminders and athletes preferred to record in the evening before they go to bed (45%) or after a training session (36%).

### Part two: focus groups and interview

#### Themes.

**Fig 8:**
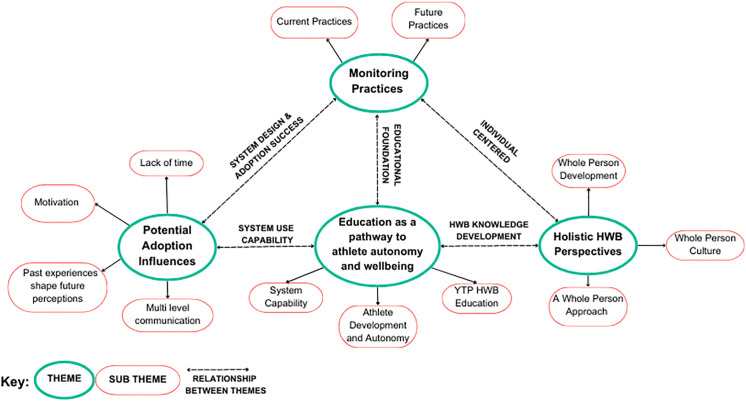
Thematic network map. Thematic network map illustrating themes, subthemes, and the interrelationship between each theme (demonstrated by the multi directional arrows).

Our analysis identified four themes: (1) *education as a pathway to athlete autonomy and wellbeing*; (2) *holistic HWB perspectives*; (3) *factors that influence adoption*; and (4) *monitoring practices* – each being representative of multiple sub-themes. Whilst each theme was developed as a standalone concept, it was also found that there were interrelationships between each theme (Fig 8).

The theme *Education as a pathway to athlete autonomy and wellbeing* is interrelated with other themes. For example, education is represented in the *Holistic HWB perspectives* theme as education contributes to HWB knowledge development. Education also supports *Factors that influence adoption by* increasing system capability, thus enhancing motivation, and relates to current *Monitoring practices* as YTP practices are built on an educational foundation and guided by a curriculum. The themes *Monitoring practices* and *Holistic HWB perspectives* are interrelated as both consider a whole person approach placing the individual at the centre of monitoring practices. Finally, the themes *Monitoring practices* and *Factors that influence adoption* are interrelated, as the effectiveness of system design and overall adoption success are shaped by the factors identified within *Factors that influence adoption.* The thematic network map (Fig 8) illustrates each theme, their subthemes and interrelationships.

***Theme 1: education as a pathway to athlete autonomy and wellbeing.*** This theme explores how education relates to: (1) the aims of a monitoring system and, (2) how education can increase capability thus driving motivation towards system adoption and implementation success. Staff identified that the primary purpose of the monitoring system is to develop athlete HWB autonomy with a secondary purpose of educating YTP staff on athlete’s HWB to enable staff to better support athlete HWB and develop intervention strategies. Both athletes and staff identified that athlete HWB education at an organisational and individual level is key to support athlete development however to do so both athletes and staff agree that developing an understanding of how to monitor and use a system is important. This theme also related to all the other identified themes. Table 2 includes illustrative quotations to support the sub-themes presented below.

**Table 2:** Subthemes and illustrative quotes relating to Theme 1: *education as a pathway to athlete autonomy and wellbeing.*

Subtheme	Data Source/illustrative Quotes
**Athlete Development and Autonomy**	**Focus Groups** “That kind of taking their ownership, that professional development of self. Who’s driving your bus? … Who’s really owning the development of what you’re doing?” **YTP Staff** “Help athletes understand all of the different factors that might influence their levels of performance, their their own well-being, and help them try and unpick” **YTP Staff** “…ultimately, that education piece is supposed to be about them understanding their own their own performance and the factors that influence that performance and health and well-being should be a central pillar of that” **YTP Staff** “So they then getting to learn about themselves, understand what’s personal to them, understand their patterns and then be able to tweak and adapt those” **YTP Staff** “Just to with a levels and stuff, helping athletes with their well-being through that as well because it is quite a lot of changes at this stage of life. So yeah, just understanding that more” **YTP Athlete**.
**YTP HWB education**	**Focus Groups** “So I guess strategically from my standpoint stuff that gives us data over a long period of time that that means we can highlight pinch points for key event groups about where injury and illness may strike and what red flags are” **YTP Staff** “England Athletics would probably be interested in knowing what’s going on with young people and making sure that their upcoming athletes are in the best possible position they can be” **YTP Athlete** “Also just to with a levels and stuff, helping athletes with their well-being through that as well because it is quite a lot of changes at this stage of life. So yeah, just [England Athletics] understanding that more” **YTP Athlete**. “Are there any common themes, common trends here that then we can start to build some coach education from an athlete education from in future years to say, well, these are markers to be aware of” **YTP Staff**
**System Capability**	**Focus Groups** “Would it also be a worthwhile experience bit for us to also complete whatever is being used as well? … to understand the process and then bits that they’re going through themselves to, if we’re going to give true feedback on it” **YTP Staff** “But I struggle to like monitor how my actual training went if that makes sense” **YTP Athlete** “I feel in in comp there’s specific things I know I need to look for, but in training, I’ve always kind of struggled finding that like fine line with it” **YTP Athlete** “Don’t quite have enough information on how to monitor it effectively” **YTP Athlete**^**a**^ “Don’t really have the tools or knowledge to do so” **YTP Athlete**^**a**^

^**a**^Open text survey response relating to survey question asking athletes (n = 24) to provide a reason as to why they do not monitor health and wellbeing.

#### Athlete development and autonomy.

Staff discussed that the primary aim of a monitoring system is to **educate athletes on their HWB** and increase their **HWB autonomy**. Staff reported that by achieving this aim, athletes will be in a better position to understand what factors affect their HWB and therefore adopt a proactive role in seeking advice specific to their needs. By adopting an athlete-led approach, staff indicate that this will increase efficiency within the YTP as staff will be more informed to deal with early intervention to help reduce injury and illness. Athletes indicated that they would like to take ownership and **autonomy of their self-development** by gaining knowledge about their own HWB.

#### YTP HWB education.

Staff identified that a secondary aim of the monitoring system should be to **educate staff on HWB prevalence** of the YTP population to be able to spot ‘red flags’ and pinch points across the programme. It was discussed that these findings could be used to educate the athletes wider support network including their personal coaches. Staff reported that due to the nature of the YTP environment, personal coaches will spend more time with their athletes and thus finding a way to support their athletes outside of the YTP environment is important and that a monitoring system provides this opportunity. Athletes also indicated that the system could help **educate the YTP on athlete HWB** to further support athlete development to help them be ‘in the best possible position they can be’.

#### System capability.

Education captures building athlete **capability in their ability to use the system**. Athletes reported that they would like to improve their HWB knowledge. 66% of athletes who do not currently monitor would like to improve their HWB, however the primary reason athletes do not monitor is because they don’t know how to and/or that they do not have the tools to do so. Staff also recognise that they will require educating on both the purpose and the use of the system to be able to assist athletes. Staff education could also aid system integration into the YTP.

***Theme 2: holistic HWB perspectives.*** This theme explores how both athletes and staff identify the holistic nature of athletic development, combining individual development outside of athletics and the holistic culture of the YTP to be able to support athlete HWB development. This theme also relates to other identified themes. Table 3 includes illustrative quotations to support the sub-themes presented below.

**Table 3:** Subthemes and illustrative quotes relating to Theme 2: *Holistic HWB perspectives.*

Subtheme	Data Source/illustrative Quotes
**A whole person approach**	**Focus Groups** “I’d normally say that if I’m happier, I compete better” **YTP Athlete** “Huge correlation between how happy an athlete is in their training environment and how well they perform**” YTP Staff** “Whether you’re, you know, socially happy, and whether you kind of have a social life that’s balanced alongside your training, is really important and how you balance that and how you maintain it but not go too crazy” **YTP Athlete** Like maybe my well-being like my mental state, like am I happy? Am I good? And all that. And then my health, Am I fit? Am I injury free? Am I ill free? Am I training well? Am I, I don’t know, am I tired all the time?” **YTP Athlete** “Yeah, I agree with the balance. I think that’s a really key factor with it. Just balancing your physical health, mental health, social health and just yeah, ensuring it all stays level. No, like dips and highs, I think that can also have a negative effect if it’s not as constant” **YTP Athlete** “I think with a lot of people it is to do with with school. Like not being able to balance training with homework and the workload that comes with school. So yeah, I think that’s probably a main factor” **YTP Staff**
**Whole person culture**	**Focus Groups** “Performance is just a part of you, it’s not you, it doesn’t define you. And when you’re not performing that you shouldn’t change you know you are still you. But I think they’ve got that the wrong way round. I think a lot of athletes have got that the wrong way round” **YTP Staff** “Like, are you actually enjoying what what you do and doing the sport. From whatever it is, whether it’s socially, [do] you actually enjoy the training, competing, don’t care what it is. But the enjoyment factor for me would be right up there because I think it takes care of 90% of the rest” **YTP Staff** “So it’s like they’re all here interested in the athletes. […] They want to make you feel good. They want to make, like, they want to help you get there” **YTP Athlete** “Are you kind of fostering those relationships because it’s important to switch off because you know, invariably you, you know, you’ll have disappointment in sport, or you’ll get injured or whatever. And it’s having that support network around you to be able to help you get through that, whether that’s, you know, parental support or, you know, having a friend like P006 on your side” **YTP Staff** “I think it’s just finding like the right people who kind of understand like they don’t have to be people who do sport, but people who understand that even though it might not look like you’re doing anything, they understand that you have to make like decisions and they might not come first all the time” **YTP Athlete**
**Whole Person Development**	“So i can live a healthier life and become a better, more fit athlete” **YTP Athlete**^**a**^ “So I can apply some of the knowledge to my own life so I can improve my own HWB which may improve my performance” **YTP Athlete**^**a**^ “Don’t quite have enough information on how to monitor it effectively” **YTP Athlete**^**b**^ “Don’t really have the tools or knowledge to do so” **YTP Athlete**^**b**^ “I will allow me to feel fresh and have enough energy for the next day” **YTP Athlete**^**c**^ “For concentration and energy” **YTP Athlete**^**c**^ “So I have the energy and motivation to train” **YTP Athlete**^**c**^ “It helps my body recover from training and allows me to function at a higher level the next day***”* YTP Athlete**^**c**^ “Essential for peak training. Poor sleep has a domino effect on other things” **YTP Athlete**^**c**^ ***“***So I feel refreshed and ready for the next session, also it improves my focus and helps muscle recovery” **YTP Athlete**^**c**^ **Focus Groups** “I don’t get sleep then I don’t want to go training and I don’t want to do anything. I just want to go back to my bed and just sleep” **YTP Athle*te*** “You can’t train well if you don’t sleep and you definitely can’t compete well if you either haven’t had the training because of the lack of sleep or on the day you haven’t had enough sleep to be able to perform the way you want to” **YTP Athlete** “It’s important that I eat enough of the right types of foods to fuel myself properly for training so I can perform to my best and reduce risk of injury” **YTP Athlete**^**d**^ “Effects performance, adds to pressure, it hurts and is bad” **YTP Athlete**^**e**^ “Affects, mood and motivation” **YTP Athlete**^**e**^

^a^Open text survey response – sample quotes related to why Athletes would like to improve their knowledge of health and wellbeing.

^b^Open text survey response – sample quotes related to why Athletes do not monitor their health and wellbeing.

^c^Open text survey response – sample quotes related to why Athletes rated sleep as a factor that effects health and wellbeing.

^d^Open text survey response – sample quotes related to why Athletes rated nutrition as a factor that effects health and wellbeing.

^e^Open text survey response – sample quotes related to why Athletes rated injury as a factor that effects health and wellbeing.

#### Whole person approach.

This theme relates to adopting/need for a **whole person approach** and recognises the complex interrelationships that form athlete HWB. Athletes and Staff understand that HWB is viewed as a holistic concept consisting of physical health, mental health and social wellbeing. Staff and athletes also understand that an individual’s HWB outside of sport can impact training and performance. When discussing HWB, athletes referred to concepts such as physical fitness, social life, and how they manage anxiety/stress inside and outside of sport.

The importance of social wellbeing to adolescent athletes was identified who related this to being able to spend time with friends which contributes to being ‘socially happy’. Further, Staff and athletes reported that when an individual feels happy inside and outside of athletics, their performance improves. Despite this, athletes are making decisions as to whether to socialise as they believe this could negatively impact on their recovery and therefore their performance as an athlete. Athletes are trying to balance demands of school, training, and social life. This finding is also observed by Staff who identify that trying to strike a balance can be problematic for athlete HWB. Athletes acknowledged that when they feel they can achieve balance between demands, this positively effects their HWB.

#### Whole person culture.

The analysis suggests that the YTP is adopting a **whole person culture** to support development of ‘healthy, happy athletes’ who find enjoyment in athletics. Athletes support this, having reported that the YTP is aware of competing demands and creates a nurturing environment to support individual HWB and achieve their athletic goals. The YTP encourages a wider athlete support network to manage difficult experiences in sport and to help reaffirm their identity beyond that as an athlete.

#### Whole person development.

Athletes shared that they would like to take ownership and autonomy of their **self-development**. They described increasing their understanding of their HWB as way to support themselves in dealing with and overcoming future challenges both as an individual and athlete.

In the online survey Athletes identified that sleep (n = 30), nutrition (n = 29), and injury (n = 21) were the main factors that affect their HWB both inside and outside of sport. See Table 2 for survey open text quotes. Athletes were asked to provide a reason for their selection and reported that sleep impact’s daily functioning and helps them ‘feel fresh’ and ‘focus in school’. Sleep also helps with recovery as an athlete and increases energy availability so that they can ‘focus and improve’ in training. Similar to sleep, athletes identified that nutrition could affect their HWB as they understand optimal nutrition is important for recovery and performance as an athlete, referring to ‘food is fuel’. Injuries have a negative effect on HWB, athletes discussed that injuries can lead to time out of sport, affect progression and future performance. Athletes reported injuries can affect mood explaining that they ‘feel upset when injured’ and motivation both inside and outside of sport thus demonstrating the interconnected relationship of the athlete and the individual. Athletes also identified positive concepts such as such self-belief and confidence which can help athletes achieve their goals.

#### Theme 3: factors that influence adoption.

Athletes and Staff identified factors that may influence system adoption with all groups drawing on their past experiences of monitoring to shape their future perceptions of monitoring systems. Both groups also identified ‘lack of time’ and ‘athlete motivation’ as adoption factors understanding that communication is important for continued use of a monitoring system. This theme also relates to other identified themes. Table 4 includes illustrative quotations to support the sub-themes presented below.

**Table 4:** Subthemes and illustrative quotes relating to Theme 3: *Factors that influence adoption.*

Subtheme	Data Source/illustrative Quotes
**Past Experiences shape future perceptions**	**Focus Groups** “Just the number, just our difficulties that we experience, with getting them to complete things. The drop off. I think it might be good, great to start, great to start with but you’ll actually just see a decline, I think” **YTP Staff** “I’ll be honest there, my memory’s terrible. So I I just probably forget and after session I’m like, I just want to go home. Like I’m especially after a hard session” **YTP Athlete** “I think I think the way in which kids are interpreting those words these days is very different to what people my age would have done a few years ago. I think the connection between well-being focus, mental health are are very clear for those young people or clearer than maybe for us” **YTP Staff** “[…] especially when I was like struggling, I tried to write it down so that then I could find out what was going well. But I just found it really difficult to just like say, oh my legs felt bad today, my legs felt bad today. Like, it didn’t go well today. It just got a bit like, this is like repetitive and boring. So, I stopped” **YTP Athlete**
**Motivation**	**Focus Groups** “I think when you see feedback it does just motivate you to keep on going or just even like the nod that what you’re doing is right. Otherwise, you’re just left a bit in the dark and you’re like, is this right? Am I doing the right thing” **YTP Athlete** “No, I guess the other thing that will perhaps help like with sort with compliance would be if if they’re getting feedback, if it’s churning out, then some useful information you know to them” **YTP Staff** “Maybe it’s even like actual sort of financial incentive we might have vouchers, or we might have discount codes or something like that” **YTP Staff** “Sometimes if you’ve had a bad session, it can be quite demotivating to write down that you felt bad. And then, especially if that goes on for a long time, it just feels like you’re in this never ending like downward slope and you just don’t want to record it. But if it’s going well then obviously, you’ll want to record it more” **YTP Athlete**
**Lack of time**	**Focus Groups** “It’s probably just a making time thing because like, I find, like, college stressful and then coming home, I just want go to bed and just chill. But I should probably add something back into my routine” **YTP Athlete** “Because obviously most of us are full time students, so sometimes we might have a piece of homework that we’ve forgotten about until the night before, and we’ve got to spend the whole night doing that, unfortunately, and then forget about doing this and then remember that we’ve got to do this and don’t want to face the consequences of they’re going to think we don’t want to be part of this England Athletics if we don’t fill this in” **YTP Athlete** “Some students, exactly as P010, describes that they’ve got that much other stuff on, and it’s just a priority outside of that moment that they’re there that they might not do it” **YTP Staff** “What more can we do within the time frame in the scope that we have and the contact that we have and with the budgets and parameters of the programme. It’s all already a big undertaking, not just for the coaches, the support staff, the College, the programme to deliver everything across the two years. It’s like, I think, is there more that we could do? Maybe. But at what cost to other elements of the programme and what budget is is there to actually collect more health and well-being data?” **YTP Staff**
**Multi-level Communication**	**Focus Groups** “Messaging and the marketing like we’ve developed a tool to help you manage health well-being, training and recovery data to maximise performance and reduce injury” **YTP Staff** “But after a couple of months, you know, I’ve seen the same results, and I feel like I’d want to talk to someone how to change to be honest” **YTP Athlete** “Talking to someone about it or just writing to someone and you know they give you a bit of feedback on it, I feel that would be very, very good” **YTP Athlete** “No, I guess the other thing that will perhaps help like with sort with compliance would be if if they’re getting feedback… Here, you know, here are some strategies or here’s where you can read more about this…If we take the time and effort to produce resources that will really, you know, connect with year 12 Year 13 students” **YTP Staff** “Maybe at the end of the month or something have a graph to see where, how your muscle soreness is like progressed or got worse. So, you can like see the results yourself” **YTP Athlete**

#### Past experiences shape future perceptions.

This theme identifies factors that influence monitoring system adoption exploring the notion that athletes and Staff draw on their **past experiences** to predict athlete monitoring behaviour. Staff referred to experiencing difficulties in obtaining information from athletes leading to a preconceived idea that system adoption will be problematic. Athletes and Staff also noted that some athletes can have poor memory recall which could impact use of a monitoring system. Athletes also drew on their past monitoring experiences – highlighting that monitoring can negatively impact their HWB in circumstances where they have repeatedly reported low mood, negative training experiences or competition results where there they feel they have underperformed, and this can **demotivate** them from future monitoring.

#### Muli-level communication.

**Multi-level communication** from Staff to athletes was also a key factor of system adoption. Staff discussed system marketing and the importance of messaging on the purpose of the system aimed at athletes to refer to injury prevention and increased performance. However, it was also discussed communicating to athletes that the monitoring system is to help develop themselves as an individual and not just an athlete’ emphasising the **whole person culture** of the YTP (discussed in Theme 2). Key relationships are also important to be able to influence the success of a system. Identifying a key staff member within the YTP who has a relationship and built trust with the athlete will assist with organisational integration of a system and help provide feedback to athletes.

HWB feedback was discussed with athletes reporting that using visuals to present their data would help them learn about their HWB. They would also like a **personal holistic approach** to feedback by being able to have conversations with a professional regarding their HWB status so that they can further develop their HWB. Staff also recognised the importance of feedback suggesting feedback in the form of educational resources. This demonstrates contradictory views from Staff and athletes in terms of the method of feedback to athletes.

#### Motivation.

**Athlete motivation** was also considered a factor of system adoption and was discussed mainly in relation athlete motivation as users of the system. Staff have indicated that athlete incentivisation, in the form of kit, nutrition and or vouchers is key to adoption. Athletes did not discuss incentivisation but instead placed importance on feedback and knowledge growth to **drive motivation**. They also reported that if they (athletes) do not receive feedback, they are likely to stop monitoring and emphasised the importance of positive feedback and guidance to help with **motivation** – ‘it’s nice to have […] maybe positive feedback in saying well done […] just to maybe give us some motivation to keep completing it or keep trying to do the best at it’. Staff also identified that asking questions that require a positive answer can also assist in athlete motivation which supports athlete findings that repeatedly recording negative items can negatively impact their HWB and system adoption.

#### Lack of time.

Both staff and athletes highlighted the potential impact of **‘lack of time’** on adoption particularly due to athlete’s school commitments. Athletes shared that school increases their stress levels thus after school, they want to relax, recover or sleep as opposed to completing another task. Staff are also aware of athlete’s school commitments and reported that the system could be seen as placing an additional burden on athletes which they are hesitant to do. Staff discussed that the YTP is also restricted on time due to its current curriculum and busy camp day schedule thus the current YTP structure does not allow for HWB monitoring.

#### System capability links with motivation and communication.

Education was also represented throughout this theme, specifically when referring to motivation and communication. This recognises that to influence adoption athletes and Staff must feel capable of being able to deliver and/or use the monitoring system effectively. By increasing their capability through knowledge generation/education, athletes and Staff may increase their self-efficacy; the belief that they can succeed delivering (Staff) or using the system to improve their HWB (athletes). In turn this could enhance their motivation to positively influence adoption. Further, finding effective ways of communicating the system relating to marketing and educational feedback is important to help increase motivation to the continued use of the system.

***Theme 4: monitoring practices.*** This theme related to current and future monitoring (including system design). This theme also relates to other identified themes. Table 5 includes illustrative quotations to support the sub-themes presented below.

**Table 5:** Subthemes and illustrative quotes relating to Theme 4: *Monitoring Practices.*

Subtheme	Data source/illustrative Quotes
**Current Practices**	**Focus Groups** “I don’t really physically monitor anything but I’m like constantly conscious, consciously thinking about like, Oh yeah, my legs are tired today, but because there is so many events, I guess it’s a bit more easy to adapt, like or do a less impactful event or something”. **YTP Athlete** “I think well after like a couple of weeks when it’s like a reoccurring thing of like, you’re always fatigued, or you can feel that something more serious is coming on. Then I’ll like say to my coach, like, I’ve been feeling like this for a couple of weeks now.” **YTP Athlete** “Just that I can like reflect back on it, like in like future train sessions or like competitions to see like my progress throughout and see that I have actually improved even if I feel like I’m not, there is like evidence that like it’s like started somewhere it’s gone to another place. And that I’m not just going nowhere”. **YTP Athlete** “But I normally like looking back on my training and sometimes I can sit there and think I’ve had a really unproductive week this week I’ve not done anything and then look back and I think, wait, I actually done quite a lot and I had a really good session Monday and yeah, the session was bad today, but I had a really good session yesterday. And then just looking back and thinking, oh well, I’ve done actually quite a bit, and it was just a day that went bad”. **YTP Athlete** “When I monitor it I just write what the training was, how hard it was, how it felt, and then what the session was.” **YTP Athlete** “I know if I’ve had a bad day and my coach can normally tell he’s just like, obviously I don’t hit a rep and then I’m just sitting there just like I’m just, I’m fed up with the training session. And then he’d be like, is it just a bad day or like, is something going on, you injured you? OK? And I’ll be like, yeah, I’m just having a bad day. Like, that’s just reset and then go again” **YTP Athlete** “It probably also indicates our curriculum, in terms of what we’re doing within nutrition and and psychology sessions”. **YTP Staff** “We have they have some one-to-one sessions in sports nutrition, sports psychology but...initially a lot of the focus will be on you know on performance. And and like the proactive and positive elements of psychology and nutrition, rather than too much problem-solving” **YTP Staff** “I don’t feel the need to” **YTP Athlete** ^a^ “Don’t quite have enough information on how to monitor it effectively” **YTP Athlete** ^a^ “Don’t really have the tools or knowledge to do so” **YTP Athlete** ^a^
**Future monitoring**	
**Individual centred**	**Focus Groups** “Everybody’s training cycles are slightly different anyway, but I actually would you know if [monitoring] doesn’t fall on a training day. I don’t think that’s a bad thing at all. It it almost again, it detaches it from it being about your training and your performance. It’s about you as an individual and how everything else is going” **YTP Staff**
**Data input**	“I just wonder whether it’s worth having a function where you can like voice note into it rather than type.” **YTP Staff** “I feel ranking how you feel like your muscle soreness on a one to five is very, it’s very vague, but I mean it’s probably the easiest way to do it. So I think having that but also maybe under each section having, do you know, additional information if you want to add something else”. **YTP Athlete**
**Monitoring frequency**	“I think a weekly thing would be great and a block or a monthly thing would be the next best thing.” **YTP Staff** “So I think to kind of take the pressure off it a little bit, I would say, yeah, three times a couple times a week is good and up to 10 minutes. We all have maybe 10 minutes, a couple times a week”. **YTP Athlete** “Ideal gold stand is something that athletes fill in daily and I can get a picture of over a longitudinal period of time, like, so it’s not, it’s not daily snapshot once a week because it doesn’t tell me a lot... Whereas I want to be able to see it, see some trends from there.” **YTP Staff** “Probably do it daily because moods change and like days change. Like if you fluctuate between each day depending on like your Wellness so it keeps like a accurate tracking of what’s gone on I suppose”. **YTP Athlete**
**Accessibility**	“You’re not going to change people’s behaviour and get them to do something that’s not app based”. **YTP Staff** “Feel like if it is something that you can do, like with your phone you can carry it anywhere with you like normally you will have your phone on you. Whereas I might not remember my training book to”. **YTP Athlete** “the only thing I would say is time and accessibility... So like whether you have your phone on you or for example, whether you need a laptop or whether you can just do it on your phone or you know whether it’s something you have to sit down and do or whether it’s something you can just do in the car. See, I would say accessibility is like the most thing for me, whereas it’s something I can literally just do on the way back from training on the way back from school if I forget maybe”. **YTP Athlete**
**Notifications**	“It pops in the timeline or the notification banner on their phone”. **YTP Staff** “I feel like I need something to be a habit before I actually go to remember doing it. I think once it’s a habit then it just becomes part of your routine. So like oh yeah, let me just do that. But until then, I think maybe having like a little notification on your phone that comes up being like reminder at a certain time.”. **YTP Athlete**

^a^Open text survey response relating to survey question asking athletes (n = 24) to provide a reason as to why they do not monitor health and wellbeing.

#### Current practices.

This theme captures the current YTP and athlete monitoring practices and explores potential future monitoring practices by considering athletes user preferences and monitoring system design. Athlete monitoring is varied and complex with athletes experiencing a heightened awareness in relation to their fatigue levels/muscle soreness, discussing that they are ‘constantly conscious’ of these factors and are making decisions about whether to adapt training sessions before seeking advice from their coach or parent.

Athletes reported using a training diary to record training session data and their mood and/or use a journal for a more reflective practice or do not recording training or HWB. Monitoring varies between event groups with endurance runners using third party apps and smart watches to assist with monitoring. This supports survey responses which identify that the most popular items recorded are training data, sleep and nutrition by using data collected via smartwatches or phone applications, emphasising that accessibility is key to monitoring.

Athlete **current monitoring practices** relates to them developing as an athlete with some athletes using monitoring to reflect on their training and mood to assist with future training and competition. Monitoring is also used as a holistic tool to bring awareness and perspective to training to increase motivation and confidence.

From an organisational perspective, Staff reported that the YTP is an educational programme, and monitoring is guided by the curriculum and data is collected to achieve the DISE qualification. However, there is autonomy amongst staff to add to the programme.

#### Future practices.

When considering **future monitoring practices** and system design, Staff acknowledged that a whole person approach should be taken. It was discussed that one of the ways in which this can be portrayed is to monitor on days when athletes are not training to emphasise to athletes that the YTP is interested in understanding their athletes as individuals. Athletes also noted that daily monitoring would be useful to ascertain HWB prevalence especially when considering items such as mood recognising that this fluctuates daily. Staff and athletes agreed that system technology must be acceptable and efficient mainly by athletes as the users of the monitoring system. There was strong consensus that the system must be app based, as this also helps with accessibility with some athletes confirming they prefer to monitor whilst travelling or before they go to sleep. Therefore, anything that couldn’t be utilised on their phones would provide an obstacle to completion. The system must be efficient to ensure action is taken by athletes. It is suggested that can be done by delivering notifications to athletes reminding them to provide their HWB information.

There were mixed views as to the frequency of data collection between all participants from daily to multiple times per week to selecting a training cycle or solely using camp days for collection. Participants have described adaptive input methods from multiple choice and shortform answers to having a voice note facility. Athletes have indicated that a multiple choice/emoji selection with the option of elaborating on their answers is preferable. Some athletes expressed concerns with not understanding scale items and therefore having the option to elaborate on their answers could address these concerns. Providing education on scales may be helpful here to improve athlete efficacy on completing the system.

## Discussion

This study represents phase one of a larger research project which used a co-creation approach to understand HWB attitudes and system design preferences. Findings indicate that athletes view monitoring HWB as either important or very important. Despite this, most athletes do not monitor their HWB, suggesting limited access to appropriate tools or insufficient knowledge to monitor effectively. Among those who do not currently monitor, 66% would like to improve their HWB knowledge. For athletes who do engage in monitoring, the most popular recorded items were training, sleep, and nutrition data. Preferred system features include mobile accessibility, adaptability, and reminder functions, while socio-environmental factors may shape system adoption. These insights will inform the design and implementation of a HWB monitoring system for the YTP. Reflexive thematic analysis generated four interrelated themes: (1) education as a pathway to athlete autonomy and wellbeing; (2) holistic HWB perspectives; (3) monitoring practices; and (4) factors that influence adoption. Collectively, the findings emphasise the importance of HWB education and whole-person development. Participants identified that the purpose of the monitoring system is to educate athletes on their own HWB and develop their autonomy. A secondary purpose of the system was to educate staff members on athlete’s HWB to be able to provide athletic support and inform the delivery of the YTP.

Adopting this approach has allowed us to improve our knowledge about Staff and athlete understanding of HWB concepts, current and future monitoring practices, system design preferences, and factors that influence the use and implementation of a proposed monitoring system. It is intended that insights and knowledge gained will be used to create a relevant, acceptable, and usable monitoring system specifically for the unique YTP environment [33].

We shall discuss our findings against each study aim.

### Study aim 1: understanding Athlete and YTP HWB concepts and monitoring practices

The findings from this study supports IOC understanding that adolescent athletic development is multidimensional [46], with a consensus across all participants that athlete HWB development is holistic; encompassing mental, physical, and social concepts. Factors outside of sport can influence athletic performance and vice versa. Considering athlete holistic development, both Staff and athletes highlighted that social wellbeing, such as time spent with friends, is important [6] and contributes to individual happiness, which may enhance athletic performance. Despite recognising the value of social wellbeing on their happiness and therefore performance, athletes reported making deliberate choices about social engagement, balancing it with the need for physical and mental recovery presenting a juxtaposition between choices they make as an athlete and that as an adolescent.

This study identifies that the YTP values a whole person development approach. A key finding is that athlete HWB education underpins the purpose of the monitoring system. Staff report the primary aim of the system is to increase athletes HWB knowledge and self-awareness empowering them to approach appropriate professionals with a clear understanding of their needs and the support they require. This finding supports literature purporting that monitoring systems can increase athlete self-awareness thus are more likely to take responsibility for their own development as individuals and athletes [22]. This approach has also been identified as a positive functional feature of a Talent Development Environment of promoting athlete personal development by providing opportunity for the athlete to act autonomously to their own development [6]. Further, the YTP promotes a wider support network to help athletes navigate challenges and reinforce their identities beyond sport. This whole-person approach helps promote athlete performance and healthy engagement in sport [46].

Athletes recognised HWB monitoring as “very important” or “important” to their training, however this study found that many athletes do not engage in monitoring practices because they lacked the knowledge and tools to do so effectively. Athletes understand that by gaining knowledge on their HWB, they will be better equipped to cope with future challenges both as an athlete and individual. For athletes who do monitor HWB, they engaged in various self-monitoring methods, including training diaries, reflective journals, or wearable technology (especially among endurance runners), although practices varied by event group. These tools were used to track not only physical metrics but also mood and motivation, offering holistic perspective on training, supporting confidence and their development as an athlete. Although studies suggest that athlete monitoring systems may not be indicative of performance success [28], this study identified that athletes believe that by being more informed about their HWB this may translate to improved performance.

While there is no formal HWB monitoring practices currently in the YTP, athletes described being ‘constantly conscious’ of their fatigue levels and muscle soreness thus adjusting their athletic training before seeking advice from their coach or parents. This demonstrates that athletes are naturally making autonomous choices over their training adaptations thus emphasising the need for HWB education. Both Staff and athletes viewed education and autonomy as components of athletic development, aligning with IOC recommendations [46].

### Study aims 2 and 3: system design preferences and factors that influence adoption

Staff discussed a user-centred design approach by prioritising the athletes’ needs with the aim of encouraging system adoption. Accessibility was a key consideration, with participants expressing a preference for completing the system via their smart phones, thus monitoring software must be compatible for use with a smart phone, supporting findings in Saw et al [25]. The system must also be supported by reminders or notifications aligning with literature on monitoring system engagement in sport [22,25]. These factors highlight the importance of system design and its compatibility with adolescence athlete’s lifestyle.

Saw et al. [29], Duignan et al. [23] and Wilson [22] report that a customised brief daily or weekly measure (assessing items such as muscle soreness, fatigue, sleep quality, stress, and mood [23,29]) supplemented with a more in-depth validated survey may be sufficient to assist the athlete to become more self-aware in their rest and recovery needs. This study found mixed preferences from both athletes and Staff regarding data collection frequency, ranging from daily inputs to less frequent with an option of utilising YTP camp days. However, athletes reported factors sensitive to change, such as mood, could be reported daily. A systematic review demonstrated that mood disturbances measured by the Profile of Mood States [47] increased with higher training load and acute fatigue during intense training periods [48]. Thus, mood monitoring can be a useful tool for coaches and athletes to inform training adjustments [48]. If regular mood monitoring is achieved, then this has the potential to support individualised optimised training load [49].

Adaptive input methods were also considered with suggestions of text, voice notes, multiple choice/emoji selection. Survey design factors are also an important consideration with some athletes expressing concerns about not understanding scale items. Providing athletes with the ability to elaborate on their answers may help address this concern; aligned to previous recommendations [25].

Literature highlights the importance of socio-environmental factors as the primary cause of system efficacy [40] however these factors tend to be discussed secondary to metric-related factors which measure reliability and validity [39]. The co-creation approach allowed for socio-environmental factors to be acknowledged before system design and implementation identifying potential factors such as perceived time constraints, prior experience with monitoring and working with adolescent athletes. Further, both Staff and athletes cited motivation and communication as important for sustained system use. In particular, feedback was cited to be a main motivation to remain engaged with the system, supporting previous literature concerning training monitoring engagement [40]. In line with findings in McGuigan et al. [50], Staff discussed the benefit of receiving implementation education on the purpose and use of the system to support athletes in system adoption and continued athlete HWB development.

This study finds variability in athlete and Staff system design preferences; however, this does not compromise the collaborative design process. In line with applied research, this study aimed to enhance understanding of athlete HWB and system design preferences, while also considering real-world implementation. A moderate level of stakeholder engagement is therefore required [51] and findings can inform key elements of the system such as useability, engagement and feedback mechanisms. These preferences can be integrated with current literature relating to practical aspects such as monitoring duration and frequency. This approach provides scope to adapt the system in future phases based on user experience, allowing it to accommodate diverse preferences and maintain a collaborative process that supports innovation.

## Study considerations

Adopting a co-creation approach to the design and implementation of the monitoring system is a key strength to enhance the system’s relevance, useability and acceptability for its intended users and supporters. This collaborative process enables early identification of any potential challenges to system adoption and implementation, helping to address the knowledge to practice gap. Using a mixed methods approach further strengthens this study. The survey provides breadth of data across the athlete cohort, whilst the focus groups add depth through contextualised insights. The alignment of qualitative and quantitative finding offers more robust findings to enhance understanding of adolescent athlete HWB and how it is monitored in a Talent Development Environment.

A limitation of this study is the sample size of athletes who completed the survey. The initial recruitment email containing the survey was sent to 242 first year athletes who, at the time of data collection, were enrolled on the YTP. We received 53 valid survey responses and 90 incomplete responses which could not be used in this study thus reducing external validity of the YTP athlete population. However, both male and female athletes and all YTP event groups was represented. Future studies could look to reduce time taken to complete the survey to potentially increase athlete participation. For the focus groups, the sprints event group was not represented for Staff or athletes as neither sprint coaches nor athletes did not respond to our invitation. Thus, findings are missing the perspectives of the sprints event group. Future studies could adopt a more targeted approach to inviting people from different event groups and invite more athletes than those who took part in the survey.

## Conclusion

This study adopted a co-creation approach to understand the HWB attitudes and design preferences to designing and implementing a monitoring system to understand the HWB of adolescent athletes enrolled in the YTP. We have provided novel insights into the views of Staff and adolescent athletes relating to HWB (physical health, mental health and social wellbeing) concepts. This study also recognises the interrelationships of these concepts on the athlete and their effect on athletic performance, which reinforces the holistic nature of athletic development.

Athlete HWB education underpins the primary purpose of a monitoring system. It aims to enhance individual understanding of their needs and the support they require. Athletes lack knowledge on how to monitor their HWB. Thus, athlete education on the purpose and use of the system is essential to enhance system user capability, motivation, and to achieve the system primary purpose.

From a practical perspective, we identified athlete monitoring preferences in terms of system accessibility, information input design, and frequency of data input to inform system design together with potential factors that influence adoption at both an organisational and individual level.

Phase two of this project is to implement the Design and Deliver aspects of the Double Diamond Framework. Findings from this study will be used to design and implement a monitoring system informed by athletes (as end users) and Staff (deliverers). This approach aims to increase the chance of system effectiveness and implementation success.

## Supporting information

S1 File Part One Athlete Survey. (PDF)

S2 File Focus Groups and Interview Top Guides – Example Questions. (PDF)
