# Supplementary material for: Monitoring health and wellbeing in adolescent track and field (athletics) athletes: A co-creation study
Source: PLoS One. 2026 Feb 27;21(2):e0341972. doi: 10.1371/journal.pone.0341972 (PMC12948129; doi:10.1371/journal.pone.0341972)
Supplement: S1 File — (PDF) [file pone.0341972.s001.pdf]

S1 File. Part One: Athlete Survey

Study 1 - Co-creation of a system to understand the health and wellbeing trends of youth athletes

---

Start of Block: Introduction

Co-creation of a system to understand the health and wellbeing of athletes in the Youth Talent Programme. Thank you for agreeing to take part in this research study. We are asking you to complete this questionnaire to understand more about your views on health and wellbeing and how you like to track your health and wellbeing.

The questionnaire is in three parts:

- **Part one** will ask for some information about yourself and your training history;
- **Part two** will ask about your views and understanding of health and wellbeing and if you currently track your health and wellbeing; and
- **Part three** will ask about how you would like to use a system that tracks your health and wellbeing.

This questionnaire should not take you longer than 25 minutes to complete. Please take your time and read each question carefully. Please provide honest and thoughtful answers and remember that there is no right or wrong answers. Before completing the questionnaire, you are required to complete a consent form (see next page). Please ensure you have read the "participant information sheet" before completing this form. You should have received this via email.

If you have any questions before starting the questionnaire please contact Natalie on nb719@exeter.ac.uk
Click the '**next**' button to get started.

End of Block: Introduction

Start of Block: Consent Form

Q1 **Consent Form**
This Consent Form is about whether you agree to taking part in the questionnaire about understanding your views on health and wellbeing and monitoring. Please read through each statement carefully to ensure your understanding.

a I confirm that I have read the information sheet dated 29/09/2024 [Version Number: 1] attached to the email with this questionnaire for the above project. I have had the opportunity to consider the information, ask questions, and have had these answered satisfactorily.

- Yes (1)
- No (2)

---

b I understand that I do not need my parents/carers to provide additional consent and/or approval for me to take part in this project, but that talking to them about my involvement is encouraged.

- Yes (1)
- No (2)

---

c I understand that my participation is voluntary and that I am free to withdraw at any time without giving any reason and without my legal rights being affected.

- Yes (1)
- No (2)

Page Break

d I understand that I can withdraw my participation at any time prior to completion of the questionnaire. I can do this by clicking exiting the survey by clicking the 'X' in the top right hand corner of each page of questions.

- Yes (1)
- No (2)

---

e I understand that if I make a request to withdraw my data after participation but before analysis has begun, then my completed questionnaire will be permanently deleted.

- Yes (1)
- No (2)

---

f I understand that if I make a request to withdraw my data after analysis then my data will be included in the analysis but will not be used for future analysis and will be deleted.

- Yes (1)
- No (2)

---

g I understand that I can request to withdraw my personal data at anytime.

- Yes (1)
- No (2)

---

h I understand that relevant sections of the data collected during the study, may be looked at by members of the research team as well as individuals from the University of Exeter and regulatory authorities for audit purposes. I give permission for these individuals to have access to my records.

- Yes (1)
- No (2)

i I understand that my name will be link-anonymised, using a unique identification code, and will not appear in any reports, articles, and/or presentations.

- ○ Yes (1)
- ○ No (2)

j I understand that data collected may be used to support other research in the future and may be shared with other researchers. If this is the case, when data is grouped or published and shared outside of the research team, no identifiable information will be provided.

- ○ Yes (1)
- ○ No (2)

k I understand that taking part involves link-anonymised survey responses to be used for the purpose of inclusion in the University's dedicated OneDrive. This means that the research team will be able to link your personal data to the survey data. Your personal data and survey data will be held on separate databases, both of which are password protected and only accessible to the research team.

- ○ Yes (1)
- ○ No (2)

l I agree for my contact details (if given) to be kept securely until the end of the project and used by researchers from the research team to contact me about: Please select both answers if you are happy to be contacted about research findings and future projects

- ▢ the findings from this project (1)
- ▢ future research projects (2)

m I agree to take part in the above project.

○ I agree (1)

○ I do not agree (2)

End of Block: Consent Form

Start of Block: Section 1 - Demographic and Training History Questions

Section 1: Background demographics and training history

The following questions ask you about your individual characteristics and training history

1 **Please provide your date of birth**

○ (DD/MM/YYYY) ____

2 **What is your sex?**

○ Male (1)

○ Female (2)

○ Prefer not to say (3)

3 **How do you describe yourself?**

○ Male (1)

○ Female (2)

○ Non-binary / third gender (3)

○ Prefer to self-describe (4) ____

○ Prefer not to say (5)

4 **Do you currently have an athletics coach?**

- ◯ Yes (1)
- ◯ No (2)
- ◯ Prefer not to say (3)

Display this question:

If Do you currently have an athletics coach? = Yes

4a **How long have you worked with your current coach?**
If you have worked with your coach for exactly 'x' number of years please insert a '0' in the 'Months' options. For example if you have been working with your coach for exactly 5 years, then type '5' in 'Years' and '0' in 'Months'

- ◯ Years ____
- ◯ Months ____

Page Break

Display this question:

If Do you currently have an athletics coach? = No

4b **How long have you not worked with a coach?**
If you have not worked with a coach for exactly 'x' number of years please insert a '0' in the 'Months' options. For example if you have not been working with a coach for exactly 5 years, then type '5' in 'Years' and '0' in 'Months'

- ◯ Years ____
- ◯ Months ____

---

5 **Please select your main Event Group**
This is the event that you spend most of your time training and competing in

- ◯ Throws (1)
- ◯ Jumps (2)
- ◯ Endurance (3)
- ◯ Sprints (4)
- ◯ Combined Events (5)

Display this question:

If Please select your main Event Group This is the event that you spend most of your time training a... = Throws

5a **Please select your primary event within the throws event group**

- ◯ Hammer Throw (1)
- ◯ Shot Put (2)
- ◯ Discuss (3)
- ◯ Javelin (4)
- ◯ Other (Please insert in text box below) (5) ________________

Display this question:

If Please select your main Event Group This is the event that you spend most of your time training a... = Jumps

5b **Please select your primary event within the jumps event group**

- ◯ Long Jump (1)
- ◯ Triple Jump (2)
- ◯ High Jump (3)
- ◯ Pole Vault (4)
- ◯ Other (please insert text in box below) (5) ________________

Display this question:

If Please select your main Event Group This is the event that you spend most of your time training a... = Endurance

5c **Please select your primary event within the endurance event group**

- ◯ 800m (1)
- ◯ 1500m (2)
- ◯ 3000m (3)
- ◯ 5000m (4)
- ◯ 10000m (5)
- ◯ Steeplechase (6)
- ◯ Race Walk (7)
- ◯ Other (please insert text in box) (8) ____

Display this question:

If Please select your main Event Group This is the event that you spend most of your time training a... = Sprints

5d **Please select your primary event within the sprints event group**

- ◯ 100m (1)
- ◯ 200m (2)
- ◯ 300m (3)
- ◯ 400m (4)
- ◯ 100m hurdles (5)
- ◯ 110m hurdles (6)
- ◯ 400m hurdles (7)
- ◯ Other (please insert text in box) (8) ____

Display this question:

If Please select your main Event Group This is the event that you spend most of your time training a... = Combined Events

5e **Please select your primary event within the combined events event group**

- ◯ Pentathlon (1)
- ◯ Heptathlon (2)
- ◯ Octathlon (3)
- ◯ Decathlon (4)
- ◯ Others (please insert text in box) (5) ____

6 **What is the highest level that you have competed in for your main Event Group?**

- ○ Club Competition (e.g. competitions organised by your local athletics league). (1)
- ○ School District Competition (e.g. competitions organised by English Schools' Athletic Association) (2)
- ○ County championships (e.g. competitions organised by county associations e.g. Nottinghamshire, Leicestershire) (3)
- ○ Area Championships (e.g. competitions where you represent your county such as Midland Counties AA, North Athletics or South of England AA) (4)
- ○ National Championships (e.g. track and field championships, cross country championships, road championships and UK School Games provided by England Athletics) (5)
- ○ International Championship (e.g. championships representing Great Britain and Northern Ireland Under 20s) (6)

---

7 **How many years have you been taking part in Athletics-specific training?**
This relates to how many years you have been taking part in Athletics and not just for your main Event Group

- ○ 1-2 years (1)
- ○ 3-4 years (2)
- ○ 5-6 years (3)
- ○ 7-8 years (4)
- ○ 9-10 years (5)
- ○ more than 10 years (6)

---

8 **How many years have you been competing in Athletics?**
This relates to how many years you have been competing in Athletics and not just for your main Event Group

- ◯ 1-2 years (1)
- ◯ 3-4 years (2)
- ◯ 5-6 years (3)
- ◯ 7-8 years (4)
- ◯ 9-10 years (5)
- ◯ More than 10 years (6)

---

9 **How many hours per week do you train for your main Event Group?**

- ◯ 1-2 hours (1)
- ◯ 3-4 hours (2)
- ◯ 5-6 hours (3)
- ◯ 7-8 hours (4)
- ◯ 9-10 hours (5)
- ◯ More than 10 hours per week (6)

---

10 **Outside of Athletics, do you train and/or compete in any other sports?**

- ◯ Yes (1)
- ◯ No (2)

Display this question:

If Outside of Athletics, do you train and/or compete in any other sports?? = Yes

10a **Please type the names of the other sports you train and/or compete in**

____

Display this question:

If Outside of Athletics, do you train and/or compete in any other sports?? = Yes

10b **How many hours per week do you train for the other sports?**
To answer this question, please add up the hours per week you spend training for all other sports (not including athletics)

- 1-2 hours (1)
- 3-4 hours (2)
- 5-6 hours (3)
- 7-8 hours (4)
- More than 8 hours per week (5)

11 **How many months of the year (12 months) do you train and compete in Athletics?**

- None (1)
- One (2)
- Two (3)
- Three (4)
- Four (5)
- Five (6)
- Six (7)
- Seven (8)
- Eight (9)
- Nine (10)
- Ten (11)
- Eleven (12)

○ Twelve (13)

---

12 **Is Athletics your main sport?**

○ Yes (1)

○ No (2)

○ Don't know (3)

---

13 **Have you dropped out of other sports to focus on Athletics?**

○ Yes (1)

○ No (2)

○ Don't know (3)

Display this question:

If Have you dropped out of other sports to focus on Athletics? = Yes

13a **How old were you when you dropped out of other sports to focus solely on Athletics?**

○ Age ____

End of Block: Section 1 - Demographic and Training History Questions

---

Start of Block: Section 2 - Health and Wellbeing

Section 2 Health and Wellbeing

When completing the rest of this questionnaire and you see the term 'health and wellbeing', we want you to think of your **physical, mental and social health and wellbeing** as an individual, not just as an athlete. Here are definitions to help you:

- **Physical** health and wellbeing means that you have no injury/illness
- **Mental** health and wellbeing means that you can cope with the normal stressors of life
- **Social** health and wellbeing means that you can maintain and build healthy relationships with family, friends, colleagues etc

1 **Below are a list of items that may affect the health and wellbeing of a youth athlete. We want to know how important each item is to you. Please consider each item and click/tap on an option to tell us how important each item is to you**.

	Very Important (6)	Important (5)	Moderately Important (4)	Somewhat Important (3)	Not important (2)	Don't know (1)
Academic pressure (1)	○	○	○	○	○	○
Body image (2)	○	○	○	○	○	○
Drinking alcohol (3)	○	○	○	○	○	○
Energy levels (4)	○	○	○	○	○	○
Family pressure (5)	○	○	○	○	○	○
Fatigue (6)	○	○	○	○	○	○
Illness (7)	○	○	○	○	○	○
Injury (8)	○	○	○	○	○	○
Mental Focus (9)	○	○	○	○	○	○
Mood (10)	○	○	○	○	○	○
Muscle soreness (11)	○	○	○	○	○	○
Nutrition (12)	○	○	○	○	○	○
Peer pressure (13)	○	○	○	○	○	○
Social Media (14)	○	○	○	○	○	○
Sleep (15)	○	○	○	○	○	○
Specialisation in sport (16)	○	○	○	○	○	○

Sports supplements (17)	○	○	○	○	○	○
Stress (18)	○	○	○	○	○	○
Training Load (19)	○	○	○	○	○	○

---

2 **Focusing on your top 3, why do you consider these the most important?** Please use the text boxes below to provide your answers for each item. Please write the item word first and then your explanation. For example: Sleep - because I feel fresh for the training the next day

○ Item 1 (1) ____

○ Item 2 (2) ____

○ Item 3 (3) ____

---

3 **Would you add any other item(s) to this list?**
Here are items that are already included in the list. We want to know if you would like to add extra items • Academic Pressure • Body image • Drinking alcohol • Energy Levels • Family Pressure • Fatigue • Illness • Injury • Mental Focus • Mood • Muscle Soreness • Nutrition • Peer Pressure • Social Media • Sleep • Specialisation in sport • Sport supplements • Stress • Training load

○ Yes (1)

○ No (2)

Display this question:

If Would you add any other item(s) to this list? Here are items that are already included in the lis... = Yes

3a **Please type what other items you would like to add**

____

Display this question:

If Would you add any other item(s) to this list? Here are items that are already included in the lis... = Yes

3b **Why are these items important to you?**
Please provide a response for each additional item you have told us about

____

4 **How important is health and wellbeing to you?**

- ◯ Very important (1)
- ◯ Important (2)
- ◯ Moderately important (3)
- ◯ Somewhat important (4)
- ◯ Not important (5)
- ◯ Don't know (6)

5 **How would you rate your current level of health and wellbeing?**

- ◯ Very good (1)
- ◯ Good (2)
- ◯ Moderate (3)
- ◯ Bad (4)
- ◯ Very bad (5)
- ◯ Don't know (6)

6 **How would you rate your current knowledge about health and wellbeing?**

◯ Very good (1)

◯ Good (2)

◯ Moderate (3)

◯ Bad (4)

◯ Very bad (5)

◯ Don't know (6)

---

7 **Would you like to improve your knowledge about health and wellbeing?**

◯ Yes (1)

◯ No (2)

◯ Don't know (3)

Display this question:

If Would you like to improve your knowledge about health and wellbeing? = Yes

7a **Why would you like to know to improve your knowledge of health and wellbeing?**

____

Display this question:

If Would you like to improve your knowledge about health and wellbeing? = No

7b **Why wouldn’t you like to improve your knowledge of health and wellbeing?**

____

---

8 **Which of the options below about health and wellbeing do you think is the most important for a youth athlete?**
Please select one answer

- ○ Physical health and wellbeing (1)
- ○ Mental health and wellbeing (2)
- ○ Social health and wellbeing (3)
- ○ They are all equally important (4)
- ○ Don't know (5)

---

9 **Do you consider monitoring your health and wellbeing an important part of your Athletic training?**
The word **'monitoring' means a way of keeping track of your health and wellbeing.** You can keep track of your health and wellbeing in a variety of ways that is meaningful to you.
For example, you can do this by writing down in a journal and/or talking to other people.

- ○ Very important (1)
- ○ Important (2)
- ○ Moderately important (3)
- ○ Somewhat important (4)
- ○ Not important (5)
- ○ Don't know (6)

---

10 **Why may monitoring health and wellbeing be helpful for a youth athlete?**
Here you can select multiple options

- ☐ To reduce injuries (1)
- ☐ To maintain performance (2)
- ☐ To prevent overtraining (3)
- ☐ To monitor the effectiveness of your training (4)
- ☐ To balance sport, education and social life (5)
- ☐ Don't know (6)
- ☐ Other (please insert answer in text box) (7) ________________

Page Break

The following questions asks you about how you monitor your health and wellbeing. The word ‘monitoring’ means a way of keeping track of your health and wellbeing. You can keep track of your health and wellbeing in a variety of ways that is meaningful to you. For example, you can do this by writing down in a journal and/or talking to other people.

---

1 **Do you currently monitor your health and wellbeing?**

- ○ Yes (1)
- ○ Sometimes (2)
- ○ No (3)

Display this question:

If Do you currently monitor your health and wellbeing? = No

1a **Why don't you monitor your health and wellbeing?**

____

Display this question:

If Do you currently monitor your health and wellbeing? = Sometimes

1b **Why do you sometimes monitor your health and wellbeing?**
Here you can select multiple options

- ☐ To reduce injuries (1)
- ☐ To maintain performance (2)
- ☐ To prevent overtraining (3)
- ☐ To monitor the effectiveness of your training (4)
- ☐ Personal development - to understand your own health and wellbeing (5)
- ☐ To help balance sport, education and social life (6)
- ☐ Other (7) ____
- ☐ Don't know (8)

Display this question:

If Do you currently monitor your health and wellbeing? = Yes

1c **Why do you monitor your health and wellbeing?**
Here you can select multiple options

- ☐ To reduce injuries (1)
- ☐ To maintain performance (2)
- ☐ To prevent overtraining (3)
- ☐ To monitor the effectiveness of your training (4)
- ☐ Personal development - to understand your own health and wellbeing (5)
- ☐ To help balance sport, education and social life (6)
- ☐ Other (7) ____
- ☐ Don't know (8)

Display this question:

If Do you currently monitor your health and wellbeing? = Yes

Or Do you currently monitor your health and wellbeing? = Sometimes

1d **Please complete the table below listing the health and wellbeing items you currently monitor.** We are interested in what you currently do to keep track of your health and wellbeing.
This can be items that you consider to be mental, physical and social health and wellbeing.
The table gives you opportunity to tell us about 10 items however if you do not record that many items, that is ok, just provide as much detail as possible for the items that you do record.
We have set out examples below to help you complete the table.

**Health and Wellbeing items currently monitored**	**How often do you monitor?**	**When do you monitor?**	**How do you monitor?**	**Why do you collect this information?**
Example 1: Sleep	*Every day*	*When I wake up*	*Use an app*	*To check that I am sleeping enough hours*
Example 2: Mood	*Once per week*	*Evening before bed*	*Write in a journal/training diary*	*To understand whether my mood is affecting my day to day activities or training*
Example 3: Session Effort	*3 x per week after each training session*	*After the session*	*Write in a journal/tell my coach*	*So that my coach can plan training and so I understand how hard or easy the session was to me*

Please note: if you are completing the questionnaire on a mobile phone, you will need to scroll down to enter this information for each item. The first 4 text boxes relate to item 1 and then you will scroll up to input information for item 2 in the next 4 boxes and so on

	Health and Wellbeing items currently monitored (1)	How often to you monitor? (2)	When do you monitor? (3)	How do you monitor? (4)	Why do you collect this information? (5)
Item 1 (1)					
Item 2 (2)					
Item 3 (3)					
Item 4 (4)					
Item 5 (5)					
Item 6 (6)					
Item 7 (7)					
Item 8 (8)					
Item 9 (9)					

Item 10 (10)					

Display this question:

If Do you currently monitor your health and wellbeing? = Yes

Or Do you currently monitor your health and wellbeing? = Sometimes

1e **Who reminds you to record information related to your health and wellbeing?**
Here you can select multiple options

- ☐ Coach (1)
- ☐ Parent (2)
- ☐ Friends/fellow athletes (3)
- ☐ No one, I do this myself (4)
- ☐ Other (5) ____

2 **Do you currently keep a training diary?**

- ○ Yes (2)
- ○ No (1)

Display this question:

If Do you currently keep a training diary? = Yes

2a **Do you use your training diary to monitor your health and wellbeing?**
Here we are interested in if you monitor your **physical training**

◯ Yes (1)

◯ No (2)

Display this question:

If Do you currently keep a training diary? = Yes

2b **What key information do you note in your training dairy?**
Here you can select multiple options

▢ How many reps and/or sets were performed during the training session (1)

▢ Distance ran/thrown/jumped. (2)

▢ Rating of Perceived Exertion/Effort level (3)

▢ How you felt before the session (4)

▢ How you felt after the session (5)

▢ How you felt during the session (6)

▢ Injury and/or illness (7)

▢ Any other information e.g. fatigue, daily stress, life events, sleep (8) ____

Display this question:

If Do you currently keep a training diary? = Yes

2c **How often do you complete your training diary?**

- ◯ Daily (1)
- ◯ 1-3 times per week (2)
- ◯ 4-6 time per week (3)
- ◯ Weekly (4)
- ◯ Monthly (5)
- ◯ Don't know (6)

Display this question:

If Do you currently keep a training diary? = Yes

2d **Each time you complete your training diary; how long does it take you?**

- ◯ 0-5 minutes (1)
- ◯ 5-10 minutes (2)
- ◯ 10-20 minutes (3)
- ◯ 20-30 minutes (4)
- ◯ More than 30 minutes (5)

Display this question:

If Do you currently keep a training diary? = Yes

2e **When do you complete your training diary?**

- ◯ In the morning when you wake up (1)
- ◯ Before a training session (2)
- ◯ After a training session (3)
- ◯ In the evening before you go to bed (4)
- ◯ Anytime of the day (5)

Display this question:

If Do you currently keep a training diary? = Yes

2f **Where do you complete your training diary?**
Here you can select multiple options

- ▢ At home (1)
- ▢ At your Athletics Club (2)
- ▢ At your School, Academy or College (3)
- ▢ Other (4) ____
- ▢ Don't know (5)

End of Block: Section 2 - Health and Wellbeing

Start of Block: Section 3 - User Preferences

Section 3: User preferences The following questions ask you about how you would like to keep track of your health and wellbeing and how you would like to use a system that tracks your health and wellbeing.

1 **How often would you be willing to provide information relating to your health, wellbeing, and training activities?**

- ◯ Daily (1)
- ◯ 1-3 times per week (2)
- ◯ 4-6 times per week (3)
- ◯ Once per week (4)
- ◯ Once per month (5)
- ◯ I would not spend any time providing this information (6)

Display this question:

If How often would you be willing to provide information relating to your health, wellbeing, and tra... = Daily

1a **How much time would you be willing to spend per day providing information about your health, wellbeing, and training activities?**

- ◯ Up to 5 minutes (1)
- ◯ Between 5-10 minutes (2)
- ◯ Between 10-20 minutes (3)
- ◯ Between 20-30 minutes (4)
- ◯ More than 30 minutes (5)

Display this question:

If How often would you be willing to provide information relating to your health, wellbeing, and tra... = 1-3 times per week

Or How often would you be willing to provide information relating to your health, wellbeing, and tra... = 4-6 times per week

Or How often would you be willing to provide information relating to your health, wellbeing, and tra... = Once per week

1b **How much time would you be willing to spend per week providing information about your health, wellbeing, and training activities?**

- ◯ Up to 5 minutes (1)
- ◯ Between 5-10 minutes (2)
- ◯ Between 10-20 minutes (3)
- ◯ Between 20-30 minutes (4)
- ◯ More than 30 minutes (5)

Display this question:

If How often would you be willing to provide information relating to your health, wellbeing, and tra... = Once per month

1c **How much time would you be willing to spend per month providing information about your health, wellbeing, and training activities?**

- ◯ Up to 5 minutes (1)
- ◯ Between 5-10 minutes (2)
- ◯ Between 10-20 minutes (3)
- ◯ Between 20-30 minutes (4)
- ◯ More than 30 minutes (5)

---

2 **On which devices are you more likely to record information about your health, wellbeing, and training activities?**
Here you can select multiple options

- ☐ Phone (1)
- ☐ Tablet (2)
- ☐ PC (3)
- ☐ Laptop (4)
- ☐ Don't know (5)

---

3 **Where would you want to record information about your health, wellbeing, and training activities?**
Here you can select multiple options

- ☐ At home (1)
- ☐ At your Athletics Club (2)
- ☐ At your School, Academy or College (3)
- ☐ Other (4) ____
- ☐ Don't know (5)

---

4 **When would you prefer to record information about your health, wellbeing, and training activities?**

- ☐ In the morning when you wake up (1)
- ☐ Before a training session (2)
- ☐ After a training session (3)
- ☐ In the evening before you got to bed (4)
- ☐ Anytime of the day (5)
- ☐ Other (6) ____
- ☐ Don't know (7)

---

5 **Would you like to receive reminders to record information about your health, wellbeing, and training activities?**

- ○ Yes (1)
- ○ No (2)
- ○ Don't know (3)

Display this question:

If Would you like to receive reminders to record information about your health, wellbeing, and train... = Yes

5a **How would you like to be reminded to record information about your health, wellbeing, and training activities?**

○ Mobile phone application e.g. WhatsApp (1)

○ Updates via email. (2)

○ Social media (e.g., YouTube, Facebook, Instagram, etc.). (3)

○ Other (4) ____

○ Don't know (5)

---

6 **Below is a list of reasons to monitor your health, wellbeing, and training activities.**
Please rank them in order of importance to you with 1 being the most important and 5 being the least important
To do this click/tap on the item and move them in order so that the item at the top of list is the most important and the item of the bottom is the least important to you

______ Reduce injuries/illness (1)
______ Maintain performance (2)
______ Prevent overtraining (3)
______ Monitor the effectiveness of your training (4)
______ Personal development - to understand your own health and wellbeing (5)

---

7 **Do you have any other reasons why you think it may be helpful to provide information about your health, wellbeing, and training activities?**
Please type your reason in the box provided

○ Yes (1) ____

○ No (2)

---

8 **Is there anything else that you think would be helpful to support the health and wellbeing of youth athletes?**
Please type your reason in the box provided

○ Yes (1) ____

○ No (2)

End of Block: Section 3 - User Preferences

Start of Block: Debrief Sheet

And finally, please answer the following questions relating to focus group opportunities, consent, use of your data and your chance to **WIN** a SportsShoes Voucher! **Focus Group Opportunity** We would like the opportunity to listen to your views and opinions on these topics and explore athlete responses to the questions in more detail. We will hold a Focus Group with 10-15 YTP Athletes who completed the questionnaire. The Focus Group will be held online at your convenience. If you would like to participate in the Focus Group please read the following and tick the box to confirm your consent. I would like to participate in a focus group with other athletes enrolled on the YTP to talk about the questionnaire responses in more detail

○ Yes (1)

○ No (2)

Prize Draw!
To show our appreciation for you completing the questionnaire, you will be entered into a prize draw whereupon 5 athletes will each have a chance to win a £20 shopping voucher. Please read the following statement and tick the box to confirm that you are happy to be entered into the prize draw. I would like to be entered into the prize draw to win one of 5 x £20 shopping vouchers. I also understand that to be entered into this prize draw, I need to provide my contact details.

○ Yes (1)

○ No (2)

Consent to contact you
Please read the following statements and tick the boxes to provide your consent for the research team to contact you about the Focus Group and the prize draw. If you are happy for us to contact you in each

scenario please tick all of the boxes. I agree for my contact details (if given) to be kept securely until the end of the project and used by researchers from the research team to contact me about:

- ☐ the findings from the project (1)
- ☐ future research projects including the Athlete Focus Group to be conducted as part of this project; and (2)
- ☐ whether I have won one of £20 shopping vouchers as part of the prize draw. (3)

The use of your data
Please read the following statement and tick the boxes to provide your final consent for us to use the data you have provided. If you are happy for your data to be used in each scenario please tick all of the boxes. I agree for my data to be used in this project and for the purposes of:

- ☐ shared with other researchers for use in future research projects (1)
- ☐ reports published in an academic publication (2)
- ☐ inclusion in an archive for a period of up to 10 years (3)

Please enter your name and email address below so we can contact you about the above. Please only provide these details if you have provided consent

- ○ Full Name ________________________________
- ○ Email Address ________________________________

Please remember to press 'submit your answers' at the bottom of this page to finish the survey

Thank you!
Thank you very much for taking part in this research study in which we aim to understand athlete's views on health and wellbeing and monitoring. This study will form part of a part of a larger study in which we aim to create a system to monitor the health and wellbeing of athletes in the Youth Talent Programme.

Withdraw Data/Participation

After participation, you can indicate that you wish to withdraw your research data at any time. If you have requested to withdraw before data analysis has begun, then your completed survey will be permanently deleted. If your request has been made after data analysis, your data will be included in the analysis but will not be used for future analysis and will be deleted. You will not have to provide a justification for wanting to withdraw and can request withdrawal of personal data at any time. Raw data will not be shared with England Athletics. Support If your participation in the study has raised any concerns about your resilience mental resilience, please contact MIND Charity on: Website: https://www.mind.org.uk/about-us/contact-us/ Tel: 0300 123 3393 Email: info@mind.org.uk Should you have any questions about the research please feel free to contact the researcher on the details below: Many thanks, Researcher: Natalie Bunce, email: nb719@exeter.ac.uk

End of Block: Debrief Sheet
