# Supplementary material for: Monitoring health and wellbeing in adolescent track and field (athletics) athletes: A co-creation study
Source: PLoS One. 2026 Feb 27;21(2):e0341972. doi: 10.1371/journal.pone.0341972 (PMC12948129; doi:10.1371/journal.pone.0341972)
Supplement: S2 File — (PDF) [file pone.0341972.s002.pdf]

**S2 File: Focus Groups and Interview Top Guides – Example Questions**

| Participants | Example Questions |
|---|---|
| **Staff** | **Part 1 Participants understanding of athlete HWB**<br>What HWB data do you currently capture?<br>Why do you measure these?<br>What other items would you like to monitor?<br>As a YTP Coach what do you need to know about the YTP athlete population to help individuals?<br>**Part 2 Understanding current monitoring practices**<br>Considering the way in which you currently collect data from athletes; what do you do which you currently value and want to continue?<br>What is missing from current system in the YTP?<br>How might the collection of data differ between event groups?<br>What are England Athletics aims for having a monitoring system in place?<br>**Part 3 How best to integrate a proposed monitoring system with the YTP**<br>From your experience with working with YTP athletes, how often do you think it is reasonable to collect data from them?<br>What do you think is the most effective way of capturing data from YTP athletes?<br>How can we integrate the system within the YTP so it forms part of the programme as opposed to a standalone requirement?<br>What do you think will be the challenges of athletes completing a HWB questionnaire?<br>How might we support athletes if they are not engaging with the system or overcome identified challenges? |
| **Athletes** | **Part 1 Participants understanding of athlete HWB**<br>What does HWB mean to you, not just as an athlete but as an individual outside of sport?<br>Do you think your HWB relates to your performance as an athlete? How/why?<br>Why do you think England Athletics is interested in understanding YTP athlete HWB?<br>**Part 2 Understanding current monitoring practices**<br>Do you monitor your HWB? Why do you monitor your HWB? / Why don't you?<br>What HWB items do you currently monitor? Why?<br>What do you currently like about the way you monitor your HWB?<br>What concerns do you have about providing HWB information?<br>**Part 3 User Behaviour Insights**<br>If you could design a monitoring system to make it easy for you to input HWB data, what would it look like? Can you give any examples of tools you currently use that you like?<br>What challenges do you think you might face when it comes to recording information about your health, wellbeing, and training activities?<br>What would help you get the most from a monitoring system? Motivation/what would you like to see in return for your effort?<br>What can England Athletics do for you to support in completing a health and well-being monitoring system? |
